# Supplementary material for: BRCA1/BARD1 ubiquitinates PCNA in unperturbed conditions to promote continuous DNA synthesis
Source: Nat Commun. 2024 May 20;15:4292. doi: 10.1038/s41467-024-48427-6 (PMC11106271; doi:10.1038/s41467-024-48427-6)
Supplement: Supplementary file 1 — Supplementary Information [file 41467_2024_48427_MOESM1_ESM.pdf]

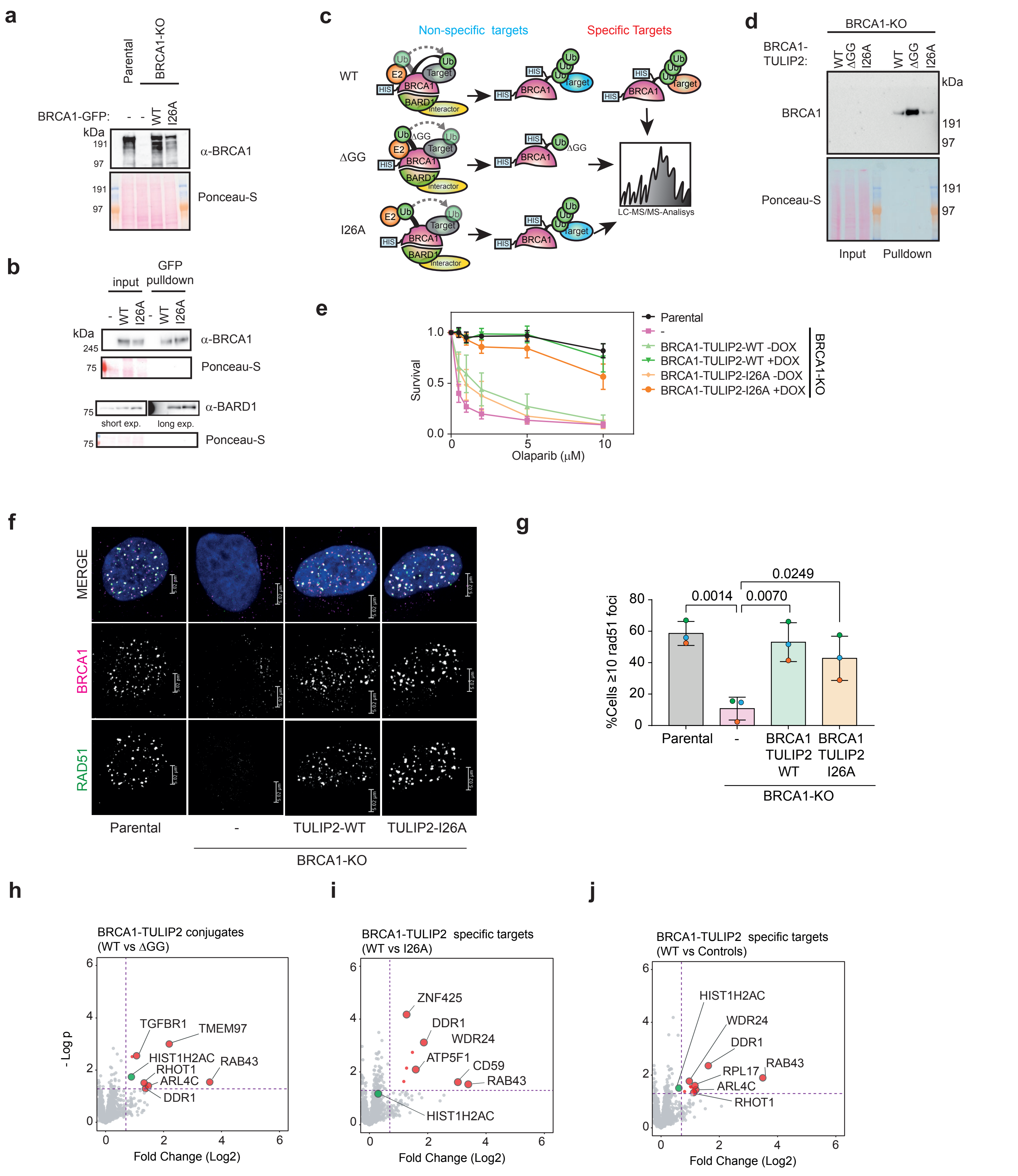

Supplementary Figure 1. BRCA1-TULIP2 Characterization. **(A).** Immunoblot showing BRCA1 levels in Parental and BRCA1-KO cells rescued or not with BRCA1-WT-GFP or BRCA1-I26A-GFP constructs from Figure 1. **(B)** Immunoblot depicting BARD1 levels co-immunoprecipitated using GFP-trap from the cells in A. **(C)** BRCA1-TULIP2 Rationale to find BRCA1 specific targets using Mass Spectrometry. **(D).** Analysis by immunoblotting of TULIP2 pull downs in BRCA1-KO cells rescue with BRCA1-TULIP2 constructs (WT, DGG and I26A). **(E).** Survival assay after treatment with Olaparib of Parental and BRCA1-KO cells rescued or not with either WT or I26A mutant BRCA1-TULIP2 constructs. Four independent experiments with 5 technical repeats were performed (N=4). Average and standard deviations are displayed. **(F-G)** Analysis by immunofluorescence against RAD51 and BRCA1 of Parental and BRCA1-KO cells rescued or not with either WT or I26A BRCA1-TULIP2 constructs. Quantification of percentage (%) of cells with equal or more than 10 RAD51 foci **(G)** and representative images **(F)** are provided. Size bars in fluorescence microscopy images represent 10 μm. Three independent experiments were performed per condition displaying the average and standard deviation (N=3). The average of each independent experiment is represented by an orange, green or purple circle. P-values correspond to two-tailed unpaired t-tests. **(H).** Volcano plot depicting statistical differences between BRCA1-WT and DGG TULIP2 constructs. Each dot represents a protein **(I)**. Volcano plot depicting statistical difference between BRCA1-WT-TULIP2 samples compared to BRCA1-I26A TULIP2 samples as controls **(J)** Volcano plot depicting statistical difference between BRCA1-WT-TULIP2 samples compared to ΔGG and BRCA1-I26A TULIP2 samples pooled together as controls. p-values conrrespond to two-tailed unpaired t-tests. Each dot represents a protein.

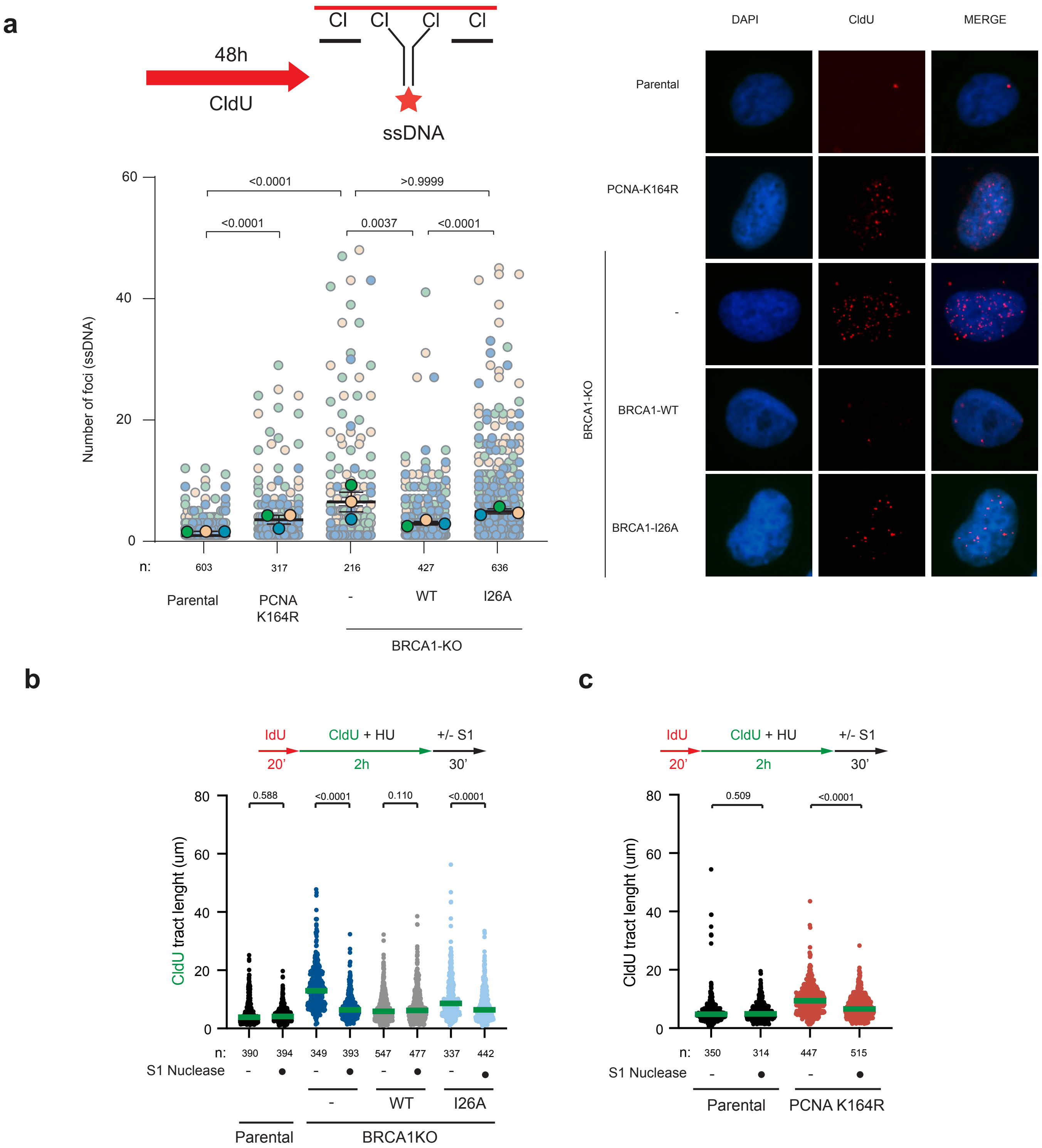

Supplementary Figure 2. Additional ssDNA gaps analysis. (A) ssDNA gaps analysys by immunofluorescence. Top: Scheme of CldU treatment for 48 h and immunostaining. Bottom: Quantification of number of ssDNA foci after CldU incubation is presented. Each circle represents a cell. Average and SEM are displayed. n: indicates the number of analyzed nuclei from 3 independent experiments. Two-tailed Kruskal-Wallis tests were performed and p-values of pair-wise comparisons are shown in the figure. Representative images are provided.**(B-C)** Formation of ssDNA gaps upon induction of mild replication stress by 0.5mM hydroxyurea in Parental, BRCA1-KO and BRCA1-WT and BRCA1-I26A rescued cells **(B)** and in Parental and PCNA-K164R mutant cells **(C)**. Top: Scheme of the IdU/CldU pulse-labelling protocol, followed by S1 nuclease treatment. Bottom: CldU tract lenghts in the indicated cell lines with and without S1 nuclease treatment. Each dot represents one fiber and the green bar represents the median. n: indicates number of measured fibers from 2 independent experiments. p values corresponding to two-tailed Mann-Whitney test are shown.

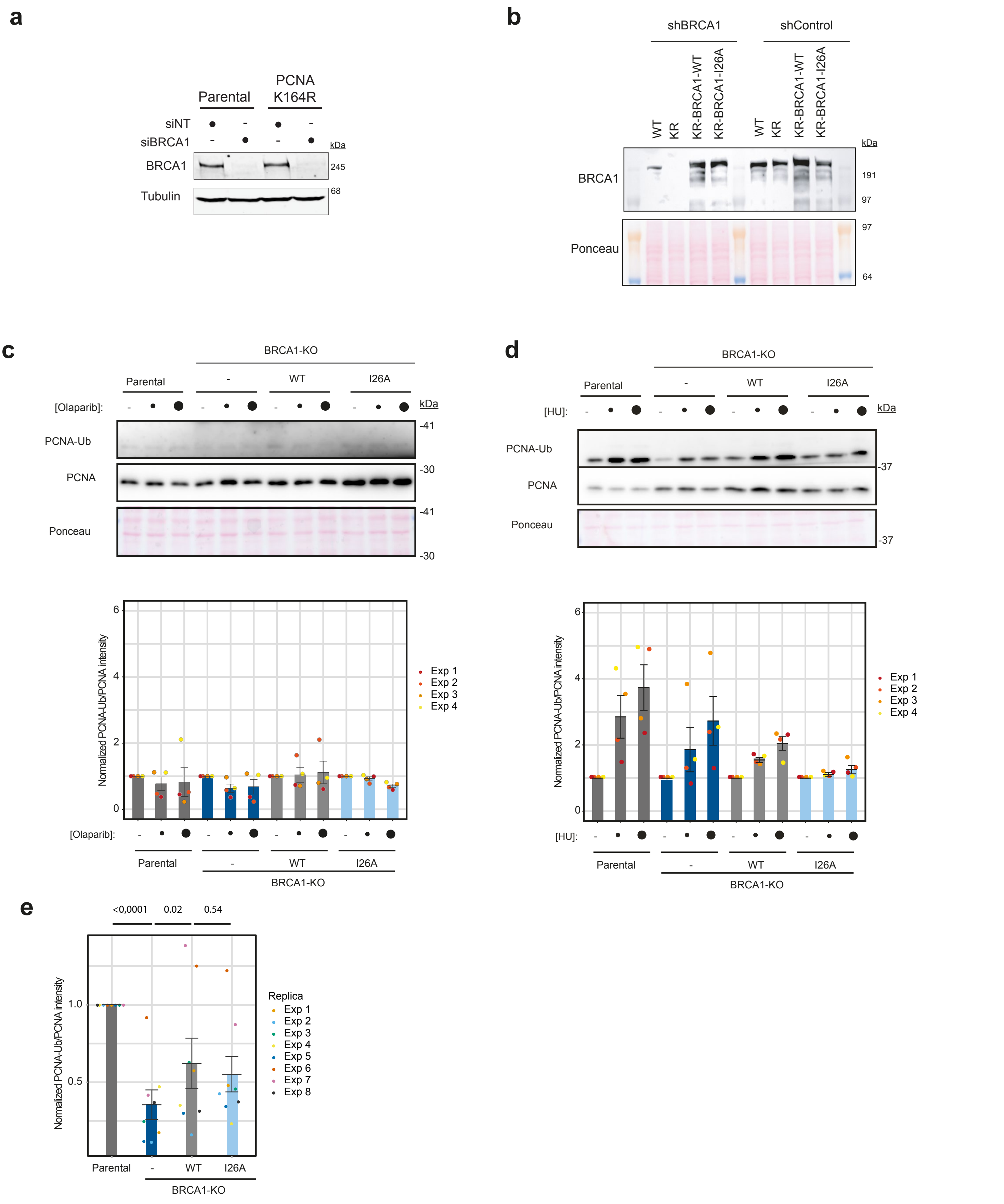

Supplementary Figure 3. PCNA Ubiquitination levels **(A)** Immunoblot showing BRCA1 levels of Parental and PCNA-BRCA1 cells after treatment with a control- or BRCA1-targeting siRNA from Figure 3A. Tubullin was used as loading control. **(B)** Immunoblot analysis of BRCA1 levels from experiemts in Figure 3 D-E **(C-D) Immunoblot analysis of endogenous PCNA ubiquitination levels after genotoxic treatments.** Parental and BRCA1-KO cells rescued or not with either BRCA1-WT-GFP or BRCA1-I26A constructs were treated for 3h with **(C)** Olaparib (0, 1μM, 10μM) or **(D)** Hydroxyurea (0, 0.5mM, 5mM), lysed and analyzed by immunoblotting. Quantification of fo PCNA-Ub/PCNA ratio normalized to untreated conditions for each cell line is provided. Each dot represents the quantification of an independent experiment. Average and SEM is depicted **(E)** Quantification of fo PCNA-Ub/PCNA levels in untreated conditions normalized to Parental levels from the experimenst in C and D. Bars represent the averages, error bars represent the SEM. Numbers above indicate p-values for two-tailed RM-Anova tests.

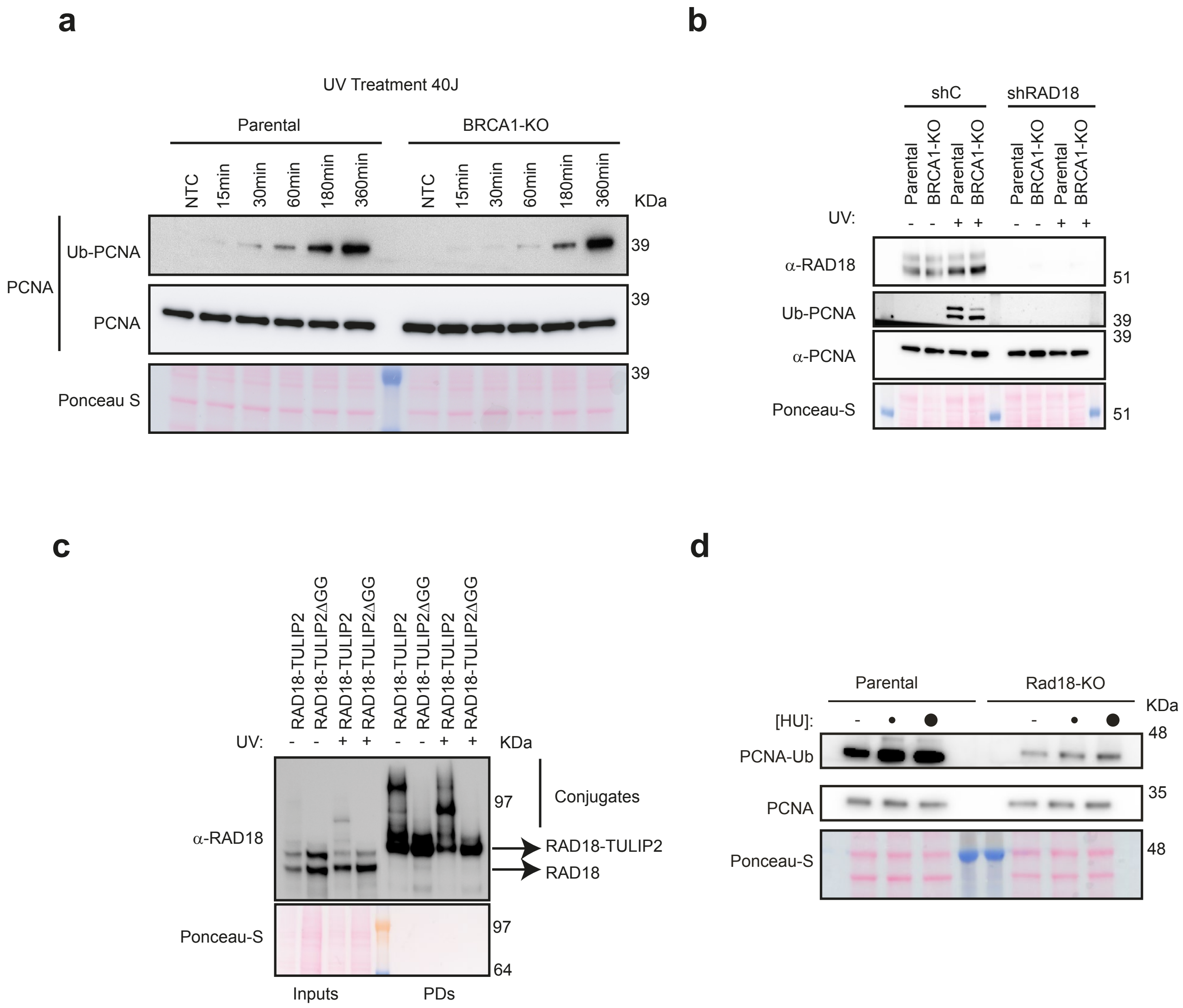

Supplementary Figure 4. RAD18 immunoblotting data. **A.** Analysis by immunoblotting of PCNA ubiquitination after 40 J/m2 UV irradiation in a time course manner in Parental and BRCA1-KO cells. **B.** Analysis by immunoblotting against PCNA 5h after 40 J/m2 UV irradiation in Parental and BRCA1-KO cell after treating with a control or RAD18-targetting shRNA. **C.** Analysis by immunoblotting of RAD18-TULIP2 samples with and without 40 J/m2 UV treatment. **D.** Analysis by immunoblotting of PCNA ubiquitination levels in Parental and RAD18-KO cells after treatment for 3h with 0, 0.5 mM or 5 mM of Hydroxyurea.

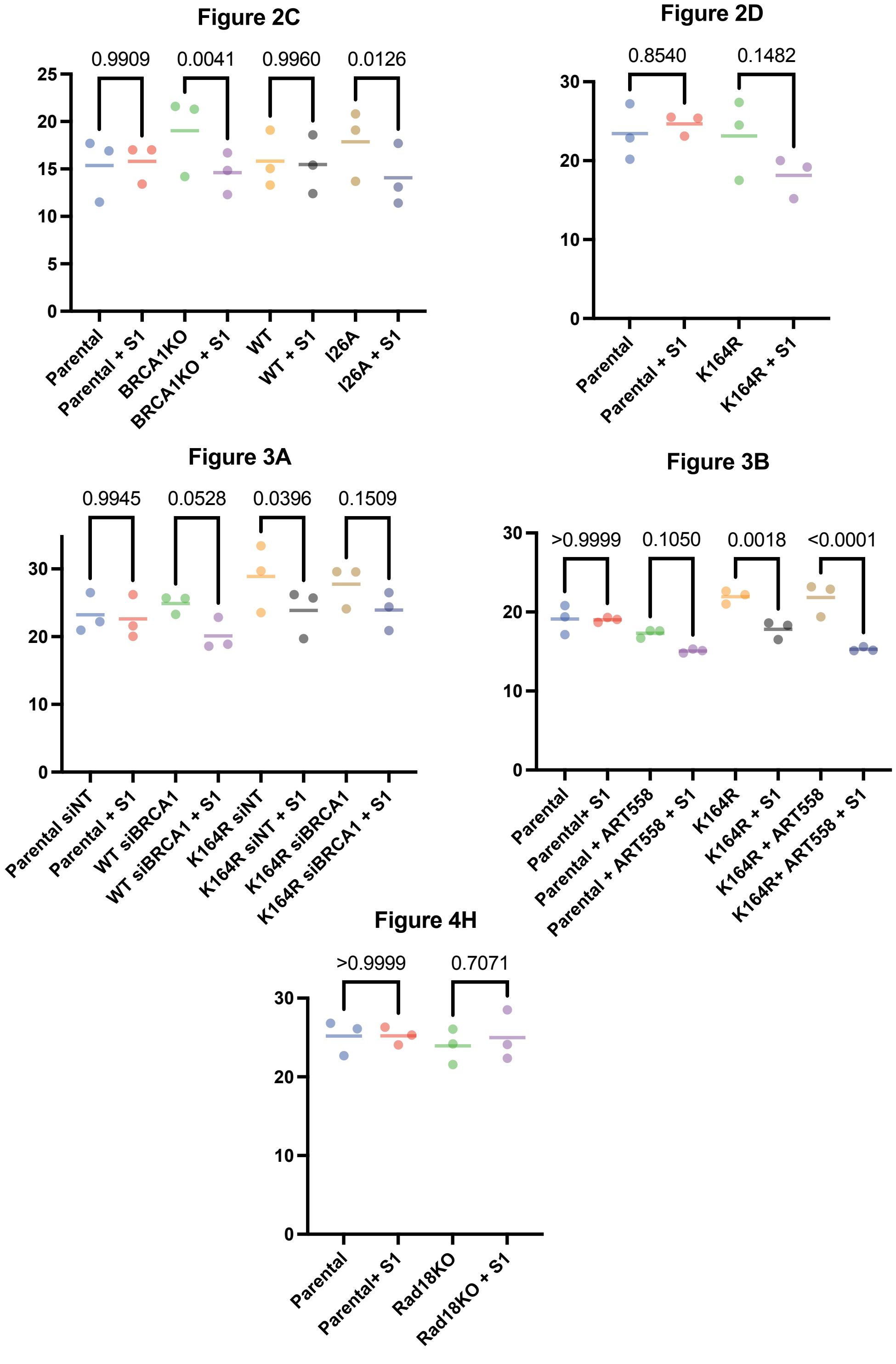

Supplementary Figure 5. Alternative statistical analysis of the different S1-fiber analysis showed in the manuscript. Repeated Measures ANOVA tests were performed using the medians of independent experiments. p - values of pairwise comparisons are indicated. Šídák method was applied for multiple testing correction.

Primer Name	Sequence	Use
BP-FW-BRCA1	GGGGACAAGTTTGTACAAAAAAGCAGGCTTCatggatttatctgctcttcgcgtt	BRCA1 TULIP2 Cloning
BP-RV-BRCA1-no-STOP	GGGGACCACTTTGTACAAGAAAGCTGGGTgtagtggcTgtgggggatct	BRCA1 TULIP2 Cloning
FW-BRCA1-I26A	GCTATGCAGAAAATCTTAGAGTGTCCCGCCTGTCTGGAGTTGATCAAGGAACCT	Introduction of I26A mutation
RV-BRCA1-I26A	AGGTTCCTTGATCAACTCCAGACAGGCGGGACACTCTAAGATTTTCTGCATAGC	Introduction of I26A mutation
BP-FW-RAD18	GGGGACAAGTTTGTACAAAAAAGCAGGCTTCatggactccctggccgag	RAD18 TULIP2 Cloning
BP-RV-RAD18	GGGGACCACTTTGTACAAGAAAGCTGGGTaattcctattacgcttgtttcttggttcaatc	RAD18 TULIP2 Cloning
RemoveStop-RAD18-FW	GCTGAGATTGAACCAAGAAACAAGCGTAATAGGAATCCAACTTTCTTGTACAAAGTTGGC	RAD18 TULIP2 Cloning
RemoveStop-RAD18-RV	GCCAACTTTGTACAAGAAAGTTGGATTCCTATTACGCTTGTTTCTTGGTTCAATCTCAGC	RAD18 TULIP2 Cloning

Supplementary Table 1: List of primers.

Antibody	Target	Dilution	Company
**Primary Antibodies**			
Rabbit anti-BARD1 (A300-263A)	BARD1	1:1000	Bethyl
Rabbit anti-BRCA1 (9010S)	BRCA1	1:1000	Cell Signaling Technology
Mouse anti-BRCA1 (OP92)	BRCA1	1:1000	Millipore
Mouse anti-BRCA1 (sc-6954)	BRCA1	1:100	Santa Cruz
Mouse anti α-Tubulin (#T9026)	α-Tubulin	1:20000	Sigma Aldrich
Mouse anti-PCNA (Sc-56)	PCNA	1:5000	Santa Cruz
Rabbit anti-RAD18 (9040S)	RAD18	1:1000	Cell Signaling Technology
Rabbit anti-RAD51 (70-001)	RAD51	1:15000	BioAcamedia
Rabbit anti-RPA1 (NA18-100UG)	RPA1	1:1000	Millipore
Mouse anti-Ubiquitin (Sc-8017)	Ubiquitin	1:1000	Santa Cruz
Rat anti-BrdU (ab6326)	BrdU/CldU	1:200 – 1:250	Abcam
anti-BrdU (#347580)	BrdU/IdU	1:250	Becton Dickinson
**Secondary Antibodies**			
HRP-conjugated Donkey anti-Rabbit (31458)	Anti-Rabbit	1:5000	Thermo Fisher Scientific
HPR-conjugated Goat antiMouse IgG (H+L) (31432)	Anti-Mouse	1:5000	Thermo Fisher Scientific
HRP-conjugated Goat anti-Rabbit (111-035-144)	Anti-Rabbit	1:5000	Jackson Immuno Research
HPR-conjugated Goat anti-Mouse IgG (H+L) (115-035-146)	Anti-Mouse	1:5000	Jackson Immuno Research
Secondary Alexa Flour 594	Anti-Rat	1:1000	Thermo Fisher Scientific
Secondary Alexa Flour 488	Anti-Rabbit	1:1000	Thermo Fisher Scientific
Secondary Alexa Flour 594	Anti-Mouse	1:1000	Thermo Fisher Scientific

Supplementary Table 2: List of antibodies. Working dilutions are indicated.
