# Supplementary material for: BRCA1/BARD1 ubiquitinates PCNA in unperturbed conditions to promote continuous DNA synthesis
Source: Nat Commun. 2024 May 20;15:4292. doi: 10.1038/s41467-024-48427-6 (PMC11106271; doi:10.1038/s41467-024-48427-6)
Supplement: Supplementary file 3 — Description of Additional Supplementary Files [file 41467_2024_48427_MOESM3_ESM.pdf]

Description of Additional Supplementary Files

File Name: Supplementary Data 1
Description: Comparative analysis results from the BRCA1-TULIP2 assay. Includes Wild Type TULIP2 and ΔGG and I26A negative controls

File Name: Supplementary Data 2
Description: Comparative analysis results from the BARD1-TULIP2 assay in Parental and BRCA1-KO cells including the ΔGG negative control

File Name: Supplementary Data 3
Description: Comparative analysis results from the BARD1-TULIP2 assay in Parental and PCNA-K164R cells.

File Name: Supplementary Data 4
Description: Comparative analysis of IPOND-MS samples from Parental, BRCA1-KO, and BRCA1-WT- and BRCA1-I26A-rescued cells.

File Name: Supplementary Data 5
Description: Comparative analysis results from the RAD18-TULIP2 assay in Parental and BRCA1-KO cells with or without treatment with UV.
