# Supplementary material for: BRCA1/BARD1 ubiquitinates PCNA in unperturbed conditions to promote continuous DNA synthesis
Source: Nat Commun. 2024 May 20;15:4292. doi: 10.1038/s41467-024-48427-6 (PMC11106271; doi:10.1038/s41467-024-48427-6)
Supplement: Supplementary file 9 — Reporting Summary [file 41467_2024_48427_MOESM9_ESM.pdf]

Corresponding author(s): Román González-Prieto
Last updated by author(s): 09/04/2024

Reporting Summary

Nature Portfolio wishes to improve the reproducibility of the work that we publish. This form provides structure for consistency and transparency in reporting. For further information on Nature Portfolio policies, see our Editorial Policies and the Editorial Policy Checklist.

Statistics

For all statistical analyses, confirm that the following items are present in the figure legend, table legend, main text, or Methods section.

n/a	Confirmed	
☐	☒	The exact sample size (n) for each experimental group/condition, given as a discrete number and unit of measurement
☐	☒	A statement on whether measurements were taken from distinct samples or whether the same sample was measured repeatedly
☐	☒	The statistical test(s) used AND whether they are one- or two-sided<br>*Only common tests should be described solely by name; describe more complex techniques in the Methods section.*
☐	☒	A description of all covariates tested
☐	☒	A description of any assumptions or corrections, such as tests of normality and adjustment for multiple comparisons
☐	☒	A full description of the statistical parameters including central tendency (e.g. means) or other basic estimates (e.g. regression coefficient) AND variation (e.g. standard deviation) or associated estimates of uncertainty (e.g. confidence intervals)
☐	☒	For null hypothesis testing, the test statistic (e.g. F, t, r) with confidence intervals, effect sizes, degrees of freedom and P value noted<br>*Give P values as exact values whenever suitable.*
☒	☐	For Bayesian analysis, information on the choice of priors and Markov chain Monte Carlo settings
☒	☐	For hierarchical and complex designs, identification of the appropriate level for tests and full reporting of outcomes
☒	☐	Estimates of effect sizes (e.g. Cohen's d, Pearson's r), indicating how they were calculated

Our web collection on statistics for biologists contains articles on many of the points above.

Software and code

Policy information about availability of computer code

Data collection	Leica LAS X 3.7.0<br>Leica LAS AF 2.7.3
Data analysis	MaxQuant 1.6.7.0<br>Perseus 1.6.7.0<br>GraphPad 10<br>MS Excel - Office 365 version<br>FiJi - ImageJ distribution (Schindelin et al. Nature Methods 9(7): 676-682)<br>DIA-NN 1.8.1

For manuscripts utilizing custom algorithms or software that are central to the research but not yet described in published literature, software must be made available to editors and reviewers. We strongly encourage code deposition in a community repository (e.g. GitHub). See the Nature Portfolio guidelines for submitting code & software for further information.

Data

Policy information about availability of data

All manuscripts must include a data availability statement. This statement should provide the following information, where applicable:

- Accession codes, unique identifiers, or web links for publicly available datasets
- A description of any restrictions on data availability
- For clinical datasets or third party data, please ensure that the statement adheres to our policy

Source data are provided with this paper in a supplementary Source data file. Any additional details are available from the corresponding author upon reasonable request.
The mass spectrometry-based proteomics data have been deposited to the ProteomeXchange Consortium via the PRIDE72 partner repository with the dataset identifier PXD039167 [https://www.ebi.ac.uk/pride/archive/projects/PXD0039167] (TULIP2 data) and PXD050319 [https://www.ebi.ac.uk/pride/archive/projects/PXD050319] (IPOND data).

Research involving human participants, their data, or biological material

Policy information about studies with human participants or human data. See also policy information about sex, gender (identity/presentation), and sexual orientation and race, ethnicity and racism.

Reporting on sex and gender	Not Applicable
Reporting on race, ethnicity, or other socially relevant groupings	Not Applicable
Population characteristics	Not Applicable
Recruitment	Not Applicable
Ethics oversight	Not Applicable

Note that full information on the approval of the study protocol must also be provided in the manuscript.

Field-specific reporting

Please select the one below that is the best fit for your research. If you are not sure, read the appropriate sections before making your selection.

☒ Life sciences ☐ Behavioural & social sciences ☐ Ecological, evolutionary & environmental sciences

For a reference copy of the document with all sections, see nature.com/documents/nr-reporting-summary-flat.pdf

Life sciences study design

All studies must disclose on these points even when the disclosure is negative.

Sample size	Sample size is indicated in each figure. The sample size was chosen to achieve an equlibrium between obtaining enough statistical power while securing the viability of the project in a resource-wise manner.
Data exclusions	No data exclusion was performed. In the statistical analysis of samples from mass spectrometry-based proteomics, proteins not identified in all replicates for at least one condition were excluded for further analysis. This is mentioned in the Methods section.
Replication	All experiments were independently replicated. The number of replicates is specified in each figure.
Randomization	Randomization was not required for any of the experiments presented because our experiments are highly quantitative and controlled. All measures have been performed by algorithms or specific software or rely in objective measures (fiber length). Thus reasearcher bias does not apply.
Blinding	Blinding was not required for any of the experiments presented because our experiments are highly quantitative and controlled. ll measures have been performed by algorithms or specific software or rely in objective measures (fiber length). Thus reasearcher bias does not apply.

Reporting for specific materials, systems and methods

We require information from authors about some types of materials, experimental systems and methods used in many studies. Here, indicate whether each material, system or method listed is relevant to your study. If you are not sure if a list item applies to your research, read the appropriate section before selecting a response.

Materials & experimental systems

n/a	Involved in the study
☐	☒ Antibodies
☐	☒ Eukaryotic cell lines
☒	☐ Palaeontology and archaeology
☒	☐ Animals and other organisms
☒	☐ Clinical data
☒	☐ Dual use research of concern
☒	☐ Plants

Methods

n/a	Involved in the study
☒	☐ ChIP-seq
☒	☐ Flow cytometry
☒	☐ MRI-based neuroimaging

Antibodies

Antibodies used

Antibodies are listed in Supplemetary Table 2
Rabbit anti-BARD1 (A300-263A) - Bethyl
Rabbit anti-BRCA1 (9010S) - Cell Signaling Technology
Mouse anti-BRCA1 (OP92) - Millipore
Mouse anti-BRCA1 (sc-6954) - Santa Cruz
Mouse anti a-Tubulin (#T9026) - Sigma Aldrich
Mouse anti-PCNA (Sc-56) - Santa Cruz
Rabbit anti-RAD18 (9040S) - Cell Signaling Technology
Rabbit anti-RAD51 (70-001) - BioAcamedia
Rabbit anti-RPA1 (NA18-100UG) - Millipore
Mouse anti-Ubiquitin (Sc-8017) - Santa Cruz
Rat anti-BrdU (ab6326) - Abcam
Mouse - anti-BrdU (#347580) - Becton Dickinson

Validation

Information from diffrerent manufacturers websites.

Antibodies

Rabbit anti-BARD1 (A300-263A) – Bethyl
- Nature (2022) 608 (7922), 413-420 DOI: 10.1038/s41586-022-05006-3
- Nat Cell Biol (2019) 21 (3), 311-318 DOI: 10.1038/s41556-019-0282-9
- Mol Cell (2021) 81 (13), 2765-2777.e6 DOI: 10.1016/j.molcel.2021.05.010
- Nat Commun (2020) 11 (1), 5007 DOI: 10.1038/s41467-020-18838-2
- Nat Commun (2019) 10 (1), 1224 DOI: 10.1038/s41467-019-09232-8

Rabbit anti-BRCA1 (9010S) - Cell Signaling Technology
- Nat Commun. 2022 Nov 7;13(1):6722. doi: 10.1038/s41467-022-34519-8
- Nat Commun 13, 6579 (2022). https://doi.org/10.1038/s41467-022-34000-6
- Nat Cancer 3, 1211–1227 (2022). https://doi.org/10.1038/s43018-022-00438-2
- Mol Cell 2021;81(15):3128-3144.e7. doi: 10.1016/j.molcel.2021.06.011.

Mouse anti-BRCA1 (OP92) – Millipore
Immunoblotting Wilson, C.A., et al. 1999. Nature Genetics 21, 236. Scully, R., et al. 1996. Science 272, 123. Immunoprecipitation, Immunofluorescence Wilson, C.A., et al. 1999. Nature Genetics 21, 236. Scully, R., et al. 1996. Science 272, 123. Frozen Sections, Paraffin Sections Wilson, C.A., et al. 1999. Nature Genetics 21, 236

Mouse anti-BRCA1 (sc-6954) - Santa Cruz
BRCA1 physically and functionally interacts with ATF1. | Houvras, Y., et al. 2000. J Biol Chem. 275: 36230-7. PMID: 10945975
BRCA1 cooperates with NUFIP and P-TEFb to activate transcription by RNA polymerase II. | Cabart, P., et al. 2004. Oncogene. 23: 5316-29. PMID: 15107825
Familial breast-ovarian cancer locus on chromosome 17q12-q23. | Narod, SA., et al. 1991. Lancet. 338: 82-3. PMID: 1676470
Linkage of early-onset familial breast cancer to chromosome 17q21. | Hall, JM., et al. 1990. Science. 250: 1684-9. PMID: 2270482
A strong candidate for the breast and ovarian cancer susceptibility gene BRCA1. | Miki, Y., et al. 1994. Science. 266: 66-71. PMID: 7545954
BRCA1 mutations in primary breast and ovarian carcinomas. | Futreal, PA., et al. 1994. Science. 266: 120-2. PMID: 7939630
Breast cancer gene offers surprises. | Nowak, R. 1994. Science. 265: 1796-9. PMID: 8091205
Localization of a breast cancer susceptibility gene, BRCA2, to chromosome 13q12-13. | Wooster, R., et al. 1994. Science. 265: 2088-90. PMID: 8091231
Nuclear redistribution of BRCA1 during viral infection. | Maul, GG., et al. 1998. Cell Growth Differ. 9: 743-55. PMID: 9751118

Mouse anti a-Tubulin (#T9026) - Sigma Aldrich
Juntao Liu et al. PLoS pathogens, 15(6), e1007817-e1007817 (2019-06-05)
Laura Y McGirt et al. The Journal of investigative dermatology, 134(4), 1101-1107 (2013-12-07)

Mouse anti-PCNA (Sc-56) - Santa Cruz
1. Smith, M.L., et al. 1994. Interaction of the p53-regulated protein Gadd45 with proliferating cell nuclear antigen. Science 266: 1376-1380.
2. Shiratsuchi, G., et al. 2015. RBM14 prevents assembly of centriolar protein complexes and maintains mitotic spindle integrity. EMBO J. 34: 97-114.

3. Mario, L.C., et al. 2016. Egg and fourth instar larvae gut of
Aedes aegypti
as a source of stem cells. Tissue Cell 48: 558-565.
4. Wu, J., et al. 2017. Effect of curcumin on glycerol-induced acute kidney
injury in rats. Sci. Rep. 7: 10114.
5. Tusi, B.K., et al. 2018. Population snapshots predict early haematopoietic
and erythroid hierarchies. Nature 555: 54-60.
6. Tobin, M.K., et al. 2019. Human hippocampal neurogenesis persists in aged
adults and Alzheimer's disease patients. Cell Stem Cell 24: 974-982.e3.
7. Rother, M.B., et al. 2020. CHD7 and 53BP1 regulate distinct pathways for
the re-ligation of DNA double-strand breaks. Nat. Commun. 11: 5775.
8. Wang, Y.Y., et al. 2021. Osteocyte exosomes accelerate benign prostatic
hyperplasia development. Mol. Cell. Endocrinol. 531: 111301.
9. Sang, S., et al. 2022. Feiyanning formula modulates the molecular
mechanism of osimertinib resistance in lung cancer by regulating the
Wnt/β-catenin pathway. Front. Pharmacol. 13: 1019451.
10.Zhou, J., et al. 2023. SP1 impacts the primordial to primary follicle tran-
sition by regulating cholesterol metabolism in granulosa cells. FASEB J.
37: e22767.
Rabbit anti-RAD18 (9040S) - Cell Signaling Technology
- J Proteomics 2022; 262:104592. doi: 10.1016/j.jprot.2022.104592.
- Life Science Alliance Jul 2021, 4 (9) e202000995; DOI: 10.26508/lsa.202000995
- J Biol Chem. 2021 Jan-Jun;296:100073. doi: 10.1074/jbc.RA120.015839.
- Nat Commun. 2020 May 1;11(1):2147. doi: 10.1038/s41467-020-16096-w.
Rabbit anti-RAD51 (70-001) – BioAcamedia
1. Nakano T et al, Homologous Recombination but Not Nucleotide Excision Repair Plays a Pivotal Role in Tolerance of DNA-Protein Cross-links in Mammalian Cells. J. Biol. Chem. 284:27065-27076 (2009) .JBC open access pdf IF (human)
2. Vaz F et al, Mutation of the Rad51C gene in a Fanconi anemia-like disorder. Nature Genetcs 42:406-409 (2010) PMID: 20400963 IF (human)
3. Nakada S. et al. RNF8 regulates assembly of RAD51 at DNA double-strand breaks in the absence of BRCA1 and 53BP1. Cancer Res. 2012 Oct 1;72(19):4974-83. WB, IF (human)
4. Shima H. et al, Activation of the SUMO modification system is required for the accumulation of RAD51 at sites of DNA damage. J Cell Sci. 126: 5284-92 (2013) PMID: 24046452 WB, IF (human)
5. Okimoto S. et al. hCAS/CSE1L regulates RAD51 distribution and focus formation for homologous recombinational repair. Genes Cells 20, 681–694, (2015) IF (human)
6. Kobayashi S. et al. Rad18 and Rnf8 facilitate homologous recombination by two distinct mechanisms, promoting Rad51 focus formation and suppressing the toxic effect of nonhomologous end joining. Oncogene. 2015 Aug 13;34(33):4403-11. IF (human, chicken)
7. Hoa N N. BRCA1 and CtIP Are Both Required to Recruit Dna2 at Double-Strand
Breaks in Homologous Recombination. PLOS one.: April 24, 2015 . PMC 4409214/ IF
(chicken)
8. Tada K. et al. Abacavir, an anti–HIV-1 drug, targets TDP1-deficient adult T cell leukemia. Science Advances 24 Apr 2015:Vol. 1, no. 3. PMID 26601161 IF (human)
9. Orthwein A et al. A mechanism for the suppression of homologous recombination in G1 cells. Nature. 2015 Dec 17;528(7582):422-6. IF (human)
10. Ning-Ang Liu. Regulation of homologous recombinational repair by lamin B1 in radiation-induced DNA damage. FASEB J. 2015 Jun;29(6):2514-25. WB, IP, IF, (human)
11.Walser F et al. Ubiquitin Phosphorylation at Thr12 Modulates the DNA Damage Response. Mol Cell. 2020 Sep 29:S1097-2765. PMID: 33022275. IF (human)
Rabbit anti-RPA1 (NA18-100UG) – Millipore
- Mansilla et al., Sci. Adv. 9, eade7997 (2023)
- Mol Cancer Ther. 2021 Sep;20(9):1561-1571. doi: 10.1158/1535-7163.MCT-20-1099.
- Cell Rep. 2018 Nov 20;25(8):2061-2069.e4. doi: 10.1016/j.celrep.2018.10.079.
- Cell Rep. 2018 Sep 25;24(13):3513-3527.e7. doi: 10.1016/j.celrep.2018.08.086.
Mouse anti-Ubiquitin (Sc-8017) - Santa Cruz
- Beaudenon, S., et al. 1999. Rsp5 Ubiquitin-protein ligase mediates DNA damage-induced degradation of the large subunit of RNA polymerase II in Saccharomyces cerevisiae. Mol. Cell. Biol. 19: 6972-6979.
- Hadweh, P., et al. 2014. The PP4R1 subunit of protein phosphatase PP4target s TRAF2 and TRAF6 to mediate inhibition of NFkB activation. Cell. Signal. 26: 2730-2737.
- Zhang, P., et al. 2015. G protein-coupled receptor 183 facilitates endothelial-to-hematopoietic transition via Notch1 inhibition. Cell Res. 25: 1093-1107.
- Naumann, C., et al. 2016. Generation of artificial N-end rule substrate proteins
in vivo and in vitro. Methods Mol. Biol. 1450: 55-83.
- Li, J., et al. 2017. Polycomb group proteins RING1A and RING1B regulate the vegetative phase transition in Arabidopsis. Front. Plant Sci. 8: 867.
- Liu, C., et al. 2018. Proteostasis by STUB1/HSP70 complex controls sensitivity to androgen receptor targeted therapy in advanced prostate cancer. Nat. Commun. 9: 4700.
- Wang, H., et al. 2019. Interplay of MKP-1 and Nrf2 drives tumor growth and drug resistance in non-small cell lung cancer. Aging 11: 11329-11346.
- Nieto, A., et al. 2020. barrestin-1 regulates DNA repair by acting as an E3-Ubiquitin ligase adaptor for 53BP1. Cell Death Differ. 27: 1200-1213.
- Nissinen, T.A., et al. 2021. Muscle follistatin gene delivery increases muscle protein synthesis independent of periodical physical inactivity and fasting. FASEB J. 35: e21387.
- Pham, D.V., et al. 2022. Adiponectin triggers breast cancer cell death via fatty acid metabolic reprogramming. J. Exp. Clin. Cancer Res. 41: 9
Rat anti-BrdU (ab6326) – Abcam

1734 publications listed. First ten in the manufacturer website:
• Adpaikar AA et al. Epithelial plasticity enhances regeneration of committed taste receptor cells following nerve injury. Exp Mol Med 55:171-182 (2023). PubMed: 36631663
• Wang YC et al. Arginine shortage induces replication stress and confers genotoxic resistance by inhibiting histone H4 translation and promoting PCNA ubiquitination. Cell Rep 42:112296 (2023). PubMed: 36961817
• Jin Z et al. Role of skeletal muscle satellite cells in the repair of osteoporotic fractures mediated by β-catenin. J Cachexia Sarcopenia Muscle 13:1403-1417 (2022). PubMed: 35178895
• Liu Z et al. Sec13 promotes oligodendrocyte differentiation and myelin repair through autocrine pleiotrophin signaling. J Clin Invest 132:N/A (2022). PubMed: 35143418
• Skovsø S et al. Beta-cell specific Insr deletion promotes insulin hypersecretion and improves glucose tolerance prior to global insulin resistance. Nat Commun 13:735 (2022). PubMed: 35136059
• Lu X et al. CBL0137 impairs homologous recombination repair and sensitizes high-grade serous ovarian carcinoma to PARP inhibitors. J Exp Clin Cancer Res 41:355 (2022). PubMed: 36539830
• Boleslavska B et al. DDX17 helicase promotes resolution of R-loop-mediated transcription-replication conflicts in human cells. Nucleic Acids Res 50:12274-12290 (2022). PubMed: 36453994
• Guerrero A et al. 3-deazaadenosine (3DA) alleviates senescence to promote cellular fitness and cell therapy efficiency in mice. Nat Aging 2:851-866 (2022). PubMed: 36438588
• Henriksson S et al. Overexpressed c-Myc Sensitizes Cells to TH1579, a Mitotic Arrest and Oxidative DNA Damage Inducer. Biomolecules 12:N/A (2022). PubMed: 36551206
• Kong C et al. Enhanced delivery of a low dose of aducanumab via FUS in 5×FAD mice, an AD model. Transl Neurodegener 11:57 (2022). PubMed: 36575534

Mouse - anti-BrdU (#347580) - Becton Dickinson

Beisker W, Dolbeare F, Gray JW. An improved immunocytochemical procedure for high-sensitivity detection of incorporated bromodeoxyuridine. Cytometry. 1987; 8:235. (Biology).
Dolbeare F, Beisker W, Pallavicini MG, Vanderlaan M, Gray JW. Cytochemistry for bromodeoxyuridine/DNA analysis: Stoichiometry and sensitivity. Cytometry. 1985; 6:521-530. (Biology).
Dolbeare F, Gratzner H, Pallavicini MG, Gray JW. Flow cytometric measurement of total DNA content and incorporated bromodeoxyuridine. Proc Natl Acad Sci U S A. 1983; 80(18):5573-5577. (Biology). View Reference
Gratzner HG. Monoclonal antibody to 5-bromo and 5-iododeoxyuridine: A new reagent for detection of DNA replication. Science. 1982; 218:474. (Biology).
Gray JW. Monoclonal antibodies against bromodeoxyuridine (special issue). Science. 1985; 6:501-673. (Biology).
Nagashima T, Hoshino T. Rapid detection of S-phase cells by anti-bromodeoxyuridine monoclonal antibody in 9L brain tumor cells in vitro and in situ. Acta Neuropathol (Berl). 1985; 66:12. (Biology).
Pinkel D, Thompson LH, Gray JW, Vanderlaan M. Measurement of sister chromatid exchanges at very low bromodeoxyuridine substitution levels using a monoclonal antibody in Chinese hamster ovary cells. Cancer Res. 1985; 45:5795. (Biology).

Eukaryotic cell lines

Policy information about cell lines and Sex and Gender in Research

Cell line source(s)

Authors thank Daniel Durocher and Sylvie Noordermeer for sharing the parental and BRCA1-KO cell lines RPE1 cell lines (1, 2), Anja Bielinsky for the Parental and PCNA-K164R RPE1 (3) cell lines , and Peter Stirling (4) for the Parental and RAD18-KO cell lines used in this work.
HEK-293T cells used for lentiviral production are from (DuBridge et al. 1987 Mol Cell Biol. 7:379-387)

1. Noordermeer, S. M., Adam, S., Setiaputra, D., Barazas, M., Pettitt, S. J., Ling, A. K., Olivieri, M., Alvarez-Quilon, A., Moatti, N., Zimmermann, M., Annunziato, S., Krastev, D. B., Song, F., Brandsma, I., Frankum, J., Brough, R., Sherker, A., Landry, S., Szilard, R. K., Munro, M. M., McEwan, A., Goullet de Rugy, T., Lin, Z. Y., Hart, T., Moffat, J., Gingras, A. C., Martin, A., van Attikum, H., Jonkers, J., Lord, C. J., Rottenberg, S., and Durocher, D. (2018) The shieldin complex mediates 53BP1-dependent DNA repair. Nature 560, 117-121
2. van de Kooij, B., Schreuder, A., Pavani, R. S., Garzero, V., Van Hoeck, A., San Martin Alonso, M., Koerse, D., Wendel, T. J., Callen, E., Boom, J., Mei, H., Cuppen, E., Nussenzweig, A., van Attikum, H., and Noordermeer, S. M. (2023) EXO1-mediated DNA repair by single-strand annealing is essential for BRCA1-deficient cells. bioRxiv, 2023.2002.2024.529205
3. Thakar, T., Leung, W., Nicolae, C. M., Clements, K. E., Shen, B., Bielinsky, A. K., and Moldovan, G. L. (2020) Ubiquitinated-PCNA protects replication forks from DNA2-mediated degradation by regulating Okazaki fragment maturation and chromatin assembly. Nature communications 11, 2147
4. Wells JP, Chang EY, Dinatto L, White J, Ryall S, Stirling PC (2022) RAD18 opposes transcription-associated genome instability through FANCD2 recruitment. PLoS genetics 18: e1010309

Authentication

The cell lines used in this study have been recently published by the indicated laboratories and no additional authentication has been performed

Mycoplasma contamination

All the cell lines in our study were tested for mycoplasma on a regular basis, being always negative.

Commonly misidentified lines
(See ICLAC register)

THe cell lines used in this study are not included in the ICLAC register.
